# Incidence of encephalopathy and comorbidity in infants with perinatal asphyxia: a comparative prospective cohort study

**DOI:** 10.3389/fped.2024.1363576

**Published:** 2024-03-27

**Authors:** Cristina Vega-del-Val, Juan Arnaez, Carlos Ochoa-Sangrador, María Garrido-Barbero, Alfredo García-Alix

**Affiliations:** ^1^ Neonatology Unit, Hospital Universitario de Burgos, Burgos, Spain; ^2^ Neonatal Neurology, Nene Foundation, Madrid, Spain; ^3^ Neonatology, Ibero-American Society of Neonatology (SIBEN), Florham Park, NJ, United States; ^4^ Department of Investigation Unit, Hospital Virgen de la Concha, Zamora, Spain; ^5^ Ciencias de la Salud, Escuela Universitaria de Enfermería, Zamora, Spain

**Keywords:** acidemia, asphyxia, perinatal, newborn, neonate, hypoxic-ischemic, encephalopathy, therapeutic hypothermia

## Abstract

**Background:**

Programs that aim to improve the detection hypoxic-ischemic encephalopathy (HIE) should establish which neonates suffering from perinatal asphyxia need to be monitored within the first 6 h of life.

**Method:**

An observational prospective cohort study of infants with gestational age ≥35 weeks, and above 1,800g, were included according to their arterial cord pH value (ApH): ≤7.00 vs. 7.01–7.10. Data was collected including obstetrical history, as well as neonatal comorbidities, including the presence of HIE, that happened within 6 h of life. A standardized neurological exam was performed at discharge.

**Results:**

There were 9,537 births; 176 infants with ApH 7.01–7.10 and 117 infants with ApH ≤7.00. All 9 cases with moderate-to-severe HIE occurred among infants with ApH ≤7.00. The incidence of global and moderate-severe HIE was 3/1,000 and 1/1,000 births, respectively. Outcome at discharge (abnormal exam or death) showed an OR 12.03 (95% CI 1.53, 94.96) in infants with ApH ≤7.00 compared to ApH 7.01–7.10 cohort. Ventilation support was 5.1 times (95% CI 2.87, 9.03) more likely to be needed by those with cord ApH ≤7.00 compared to those with ApH 7.01–7.10, as well as hypoglycemia (37% vs. 25%; *p* = 0.026). In 55%, hypoglycemia occurred despite oral and/or intravenous glucose administration had been already initiated.

**Conclusions:**

Cord pH 7.00 might be a safe pH cut-off point when developing protocols to monitor infants born with acidemia in order to identify infants with moderate or severe HIE early on. There is non-negligible comorbidity in the ApH ≤7.00 cohort, but also in the 7.01–7.10 cohort.

## 1 Introduction

Therapeutic hypothermia (TH) is the standard treatment in most developed countries for newborns with moderate or severe hypoxic ischemic encephalopathy (HIE) (1). However, from the evidence derived on the benefit of this therapy from the first clinical trials, there is concern about how to improve its effectiveness at the time of application in clinical practice (2–6).

The therapeutic window is very short and delay of initiation of hypothermia may influence neurological outcome. Thus, a key aspect of any hypothermia program is awareness and timely identification of infants at risk for encephalopathy (5, 7).

One of the most consistent eligibility criteria set in the clinical trials included a pH of ≤7.00 in a sample of umbilical cord blood, as the most objective assessment of the presence of intrapartum hypoxia-ischemia (8–13). In fact, identification and prevention of real acidemia is the aim of intrapartum fetal monitoring. There is a consensus on ascribing cerebral palsy to intrapartum hypoxia states, which holds that arterial pH should be ≤7.00 (14), while severe acidemia is associated with HIE and long-term adverse outcomes (15, 16). However, though pH ≤7.00 seems to a reasonable threshold, most published series have included cooled infants diagnosed with moderate or severe HIE with pH above 7.00 (17–26).

Programs that aim to improve the detection of newborns with HIE should establish which neonates suffering from perinatal asphyxia need to be strictly monitored within the first 6 h of life in order to decide whether to administer TH. To our knowledge there are scarce studies that have examined the incidence of HIE and comorbid factors within 6 h of life in a prospective cohort of newborns with perinatal acidemia.

This knowledge is especially relevant in neonatal intensive care units (NICUs) with high rates of admission, since programs designed to monitor candidate infants for TH may include serial neurological examinations as well as permit the detection of comorbid factors (e.g., hypoglycemia, hypotension, and others) (27–32). Furthermore, the advantages of narrow monitoring have to be weighed against the emotional implications of the separation of the infants from their parents as well as the interruption of skin-to-skin care.

The aim of this prospective cohort study was to compare the prevalence of HIE and other comorbidities within the first six hours of life, and the neurological status at discharge of two cohorts of newborns with perinatal acidosis (arterial cord pH ≤7.00 vs. 7.01–7.10).

## 2 Method

### 2.1 Study design

This was an observational cohort study using data prospectively entered in a database, from all consecutive liveborn infants, with gestational age at birth 35 weeks or greater, and above 1,800g, delivered in a university teaching hospital. This center is a referral hospital for HIE patients, and this research is part of a broader multicenter project designed to improve the identification of HIE and care for newborns with perinatal asphyxia (33–35).

Two cohorts of infants were included according to their arterial cord pH value (ApH) at birth: (A) infants with ApH between 7.01 and 7.10 born from June 1, 2011 to June 1, 2013; and (B) infants with ApH ≤7.00 born from June 1, 2011 to June 1, 2015. Recruitment of the cohort with ApH ≤7.00 was two years longer than it was for cohort pH 7.01–7.10 in order to include a larger number of patients with the inclusion criteria in accordance with a statistical power model.

The policy of the maternity unit is to take paired umbilical cord blood samples in all babies in the delivery room. Immediately after delivery, a segment of umbilical cord is isolated between cord clamps. At the time of the study early cord clamping (less than 30 s after birth) was performed. The umbilical artery and vein were serially punctured with a 14-gauge needle and the blood was collected into a pre-heparinized capillary tube by capillary action. The blood gas values were analyzed within 15 min of delivery. Of the total of 9,537 births in the period, pH samples from 176 infants could not be obtained at birth, and in 12 infants these only could be obtained from one vessel. In the rest both cord samples were extracted. The blood gas values used were those obtained directly by the gasometer without further corrections (i.e., correction of the pH value depending on the partial pressure pCO_2_). To prevent lost patients and to avoid errors, the pediatric and obstetric departments had their own birth databases, and the two were merged.

The protocol for the cohort of infants with ApH ≤7.00 included admission to the neonatal unit, while the protocol for the cohort of infants with ApH 7.01–7.10 did not include admission. These infants were left with their parents in the delivery room unless there was clinical instability. Both cohorts underwent clinical examinations and capillary glucose determinations within 6 h of life. Hypoglycemia was defined as glucose value <46 mg/dl (29, 30, 32).

Breastfeeding was started immediately after birth in infants with ApH 7.01–7.10 if the infant remained stable, while in the cohort of infants with ApH ≤7.00 the decision was made at the discretion of the physician at bedside according to hemodynamic and respiratory status and gas values. The standard protocol for the use of intravenous glucose was applied when the oral route was not advisable due to altered neurological or cardiorespiratory status or high lactic acidosis, and/or glucose levels persistently altered despite oral feeding.

Infants diagnosed with pathologies that may indicate a birth with perinatal asphyxia or that mimic HIE were excluded: major congenital malformations, genetic or chromosomal syndrome, congenital metabolic disorder, neuromuscular disease or spinal cord injury, and neuroimaging findings suggestive of prenatal brain damage.

The clinical protocol established to perform neurological exams at three times points (1–2, 3–4, and 5–6 h of life) and the severity of HIE were graded as mild, moderate, or severe according to a previously reported modified Sarnat score (36). An educational program on the assessment and interpretation of the different items included in the HIE score for all 5 staff members and 12 residents that participated in the study was carried out before recruiting infants.

We did not assess the amplitude integrated electroencephalography (aEEG) items included in that score as infants with cord pH 7.01–7.10 stayed with their parents and were not aEEG-monitored. A final score of HIE was determined for each infant by the physician at bedside using the most recent exam performed within 6 h of life, or the latest exam before the indication that TH was achieved. Infants diagnosed with moderate or severe HIE were treated with servo-controlled whole-body TH following national guidelines (37).

A neurological exam was performed at discharge which included craniofacial features, alertness, cranial nerves, motor items (muscular tone, strength, myotatic reflexes, general movements), and behavior (transition between states and interest in visual stimuli).

Extensive data from each cohort was prospectively collected in the database including maternal, obstetrical and perinatal history: control during pregnancy, pregnancy disease (hypertension, diabetes mellitus, thyroid disorders, congenital infections), intrauterine growth retardation, mode of delivery, delivery presentation, maternal anesthesia, sentinel event, need for resuscitation, and Apgar scores. Comorbidities within 6 h of life were included: the need for respiratory support (ventilation and/or need for oxygen), use of inotropes, volume expansion or transfusion, hypoglycemia, and use of glucose therapy.

An informed consent explaining what the project consisted of was requested from parents, and the study was approved by the research ethics committee of the participating hospital (CEIC 916).

### 2.2 Statistical analysis

As periods of recruitment differed in the two cohorts, calculated incidence for the entire cohort of infants with pH ≤7.10 as well as for those with 7.01–7.10 was in relation to the period of June 2011 to May 2013, and for those infants with pH ≤7.00 it was in relation the entire period of June 2011 to December 2015.

Descriptive analysis (absolute and relative frequencies for qualitative variables and measures of central tendency for quantitative variables) was made for all variables.

Differences between the two cohorts were evaluated with the Chi-square test, analysis of variance (ANOVA), Mann-Whitney *U*-test, or Kruskal-Wallis test, as appropriate. Logistic regression analysis was used to examine the relationship between perinatal variables and the association between the study cohorts and the outcome, glycemic values, and clinical variables.

*P* < 0.05 was considered to be statistically significant. Data were analyzed using SPSS version 21 (IBM, NY, USA).

## 3 Results

**Figure 1:**
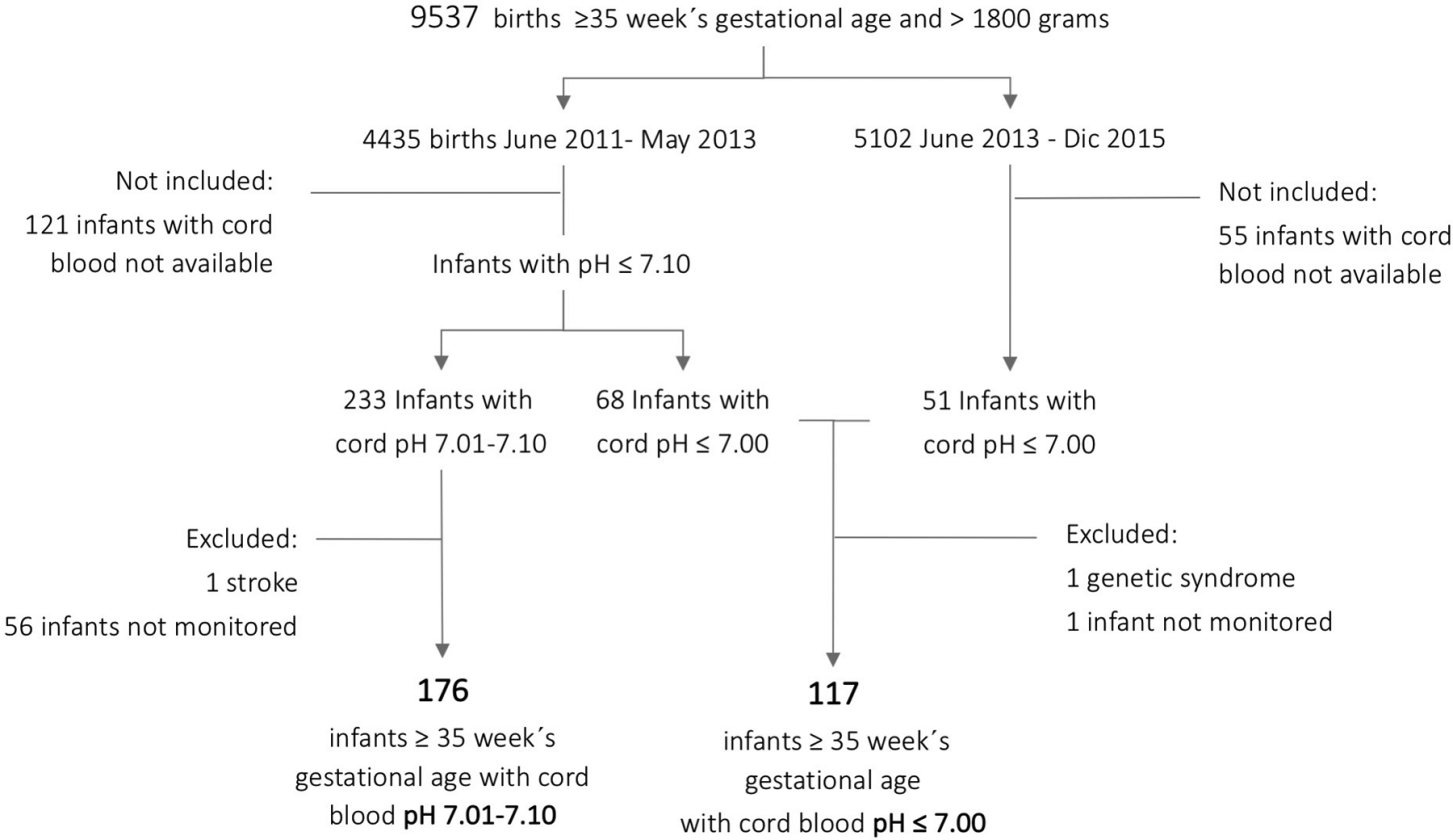
Flow chart of infant recruitment.

Three hundred fifty-two infants ≥35 weeks gestational age and >1,800g comprised the entire cohort, 119 with ApH ≤7.00 and 233 with ApH 7.01–7.10. This represented a global incidence of 67.8/1,000 births with ApH ≤7.10: 12.4/1,000 for those with ApH ≤7.00 and 52.5/1,000 for infants with pH 7.01–7.10. After excluding those infants who were not prospectively monitored or had an underlying disease, the final cohort for analysis comprised 176 infants with ApH 7.01–7.10 and 117 infants with ApH ≤7.00 (Figure 1).

Table 1 summarizes obstetrical and perinatal variables of the two cohorts. There were no differences in pregnancy-related variables, but there were regarding perinatal ones. Infants with ApH ≤7.00 had more emergency caesarean deliveries, fewer cephalic presentations, more abnormal fetal heart rate and sentinel events, more chorioamnionitis, more maternal general anesthesia, greater need for resuscitation, and lower Apgar scores at one and five minutes, compared to infants with ApH 7.01–7.10 (*p* = 0.001).

**Table 1:** Obstetric and perinatal characteristics of the cohort study.

	Total cohort (*N* = 293)	Cohort A pH 7.01–7.10 (*N* = 176)	Cohort B pH ≤7.00 (*N* = 117)	*P*-value	OR (95% CI)
Maternal age, years	33 (30, 37)	33 (30, 36)	34 (31, 37)	0.119	
Maternal age >40 years	29/292 (10)	20/176 (11)	9/116 (8)	0.314	0.68 (0.32, 1.45)
Well controlled pregnancy	289/293 (99)	174/176 (99)	115/117 (98)	1.000	0.99 (0.97, 1.02)
Hypertension or diabetes	30/293 (10)	16/176 (9)	14/117 (12)	0.427	1.32 (0.67, 2.59)
Hypothyroidism	26/293 (9)	15/176 (9)	11/117 (9)	0.796	1.10 (0.53, 2.32)
Multiple pregnancy	12/293 (4)	7/176 (4)	5/117 (4)	0.535	
Primiparity	154/293 (53)	92/176 (52)	62/117 (53)	0.904	1.01 (0.81, 1.27)
Rupture of membranes, hours	5 (1, 12)	6 (2, 12)	5 (1, 11)	0.091	
Rupture of membranes >18 h before birth	36/287 (13)	25/174 (14)	11/113 (10)	0.247	0.68 (0.35, 1.32)
Chorioamnionitis	5/286 (2)	0/175	5/111 (5)	0.008	
Gestation age, weeks	40 (38, 40)	40 (38, 40)	40 (39, 40)	0.943	
Male	135/293 (46)	85 (48)	50 (43)	0.350	1.11 (0.90, 1.37)
Birth weight, g	3,146 ± 485	3,167 ± 436	3,112 ± 551	0.343	
Birth length, cm	49.9 ± 2.1	50.1 ± 1.9	49.8 ± 2.5	0.389	
Birth head circumference, cm	34.1 ± 1.5	34.3 ± 1.4	33.9 ± 1.6	0.035	
Weight <2DE for GE	12/292 (4)	6 (3)	6 (5)	0.551	1.52 (0.50, 4.59)
Emergency caesarean	56/293 (19)	19/176 (11)	37/117 (32)	0.000	2.87 (1.79, 4.60)
Spontaneous labor	131/293 (45)	93/176 (53)	38/117 (33)	0.001	0.61 (0.46, 0.83)
Cephalic presentation	271/293 (93)	168/176 (96)	103/117 (88)	0.018	0.92 (0.86, 0.99)
General anesthesia	7/292 (2)	1/176 (1)	6/116 (5)	0.017	9.10 (1.11, 74.64)
Abnormal fetal heart rate	201/279 (72)	118/175 (67)	83/104 (80)	0.046	
Sentinel event	4/292 (1)	0/176	4/116 (3)	0.024	
Meconium-stained amniotic fluid	81/292 (28)	45/176 (26)	36/116 (31)	0.307	1.21 (0.84, 1.76)
1-min Apgar score ≤5	53/292 (18)	12/175 (7)	41/117 (35)	0.000	5.11 (2.81, 9.30)
5-min Apgar score ≤5	18/292 (6)	5/175 (3)	13/117 (11)	0.004	3.89 (1.42, 10.62)
Advanced resuscitation^a^	19/292 (7)	2/175 (1)	17/117 (12)	0.000	12.71 (2.99, 54.00)
Cord blood gas					
pH (UA)	7.01 ± 0.07	7.06 ± 0.03	6.94 ± 0.06	0.000	
pH (UV)	7.12 ± 0.11	7.17 ± 0.73	7.04 ± 0.10	0.000	
Bic, mmol/L (UA)	16.4 ± 4.5	17.24 ± 4.27	15.00 ± 4.65	0.000	
Base deficit, mmol/L (UA)	14.7 ± 3.9	13.48 ± 3.42	16.59 ± 3.87	0.000	
Base deficit ≥16 (UA)	103/277 (37)	40/170 (24)	63/107 (59)	0.000	2.50 (1.83, 3.42)
PaCO_2_, mmHg (UA)	63.6 ± 17.6	60.36 ± 14.97	72.44 ± 20.94	0.000	
PaO_2_, mmHg (UA)	12.3 ± 5.6	12.88 ± 5.61	10.88 ± 5.23	0.017	

Categorical variables are expressed in *n*/*N* (%). Quantitative variables are expressed with mean ± standard deviation. GA, gestational age; RR, relative risk; UA, umbilical artery; UV, umbilical vein.

^a^ Advanced resuscitation: intubation, chest compressions, and/or epinephrine.

### 3.1 Neonatal encephalopathy within 6 h of life and exam at discharge

There were no differences in the number of exams performed on each infant between the two cohorts in order to assess the presence and severity of NE: a median of 3 times (IQR 3,3). Twenty-nine infants (10%) were diagnosed with HIE during the study period: 20 had mild HIE, 6 moderate HIE, and 3 severe HIE. As all cases with moderate-to-severe HIE occurred among infants with ApH ≤7.00, incidence was 7.6%. The incidence of global and moderate-severe NE considering the total number of births in the period 2011–2015 was 3/1,000 and 1/1,000 births, respectively. All 9 infants received TH; hypothermia was initiated in all cases before 6 h of life, reaching the target core temperature of 33°C–34°C at a median age of 1 h (IQR 1,3).

The two infants who died had ApH ≤7.00. Among surviving infants, 7 had altered exams at discharge, 6 of them (5%) from the cohort of infants with ApH ≤7.00 (*p* = 0.009). The remaining patient was a newborn with ApH of 7.08 with undetectable glycemia at birth, along with hyperlactatemia and hyperammonemia. This infant had intense irritability with hyperreflexia within 6 h of life, and she was categorized as mild HIE. She presented progressive analytical improvement, debuted with seizures, and in the MRI showed global atrophy. At discharge she showed clinical signs of hyperexcitability with cramped-synchronized general movements, increased muscle tone, and myotatic reflexes. Blood, urine, and cerebrospinal infectious and metabolic studies, as well as muscle and skin biopsy, were normal.

Regarding outcome at discharge (abnormal exam or death) infants with ApH ≤7.00 showed an OR of 12.03 (95% CI 1.53, 94.96; *p* = 0.003) compared to those with ApH 7.01–7.10.

### 3.2 Other comorbidities within 6 h after birth

Nearly all infants (98%) with ApH ≤7.00 were admitted at birth to the neonatal unit in accordance with the protocol. Infants with ApH 7.01–7.10 stayed with the parents unless there was clinical instability: 35 out of 176 (20%) infants required admission within the first 6 h.

**Figure 2:**
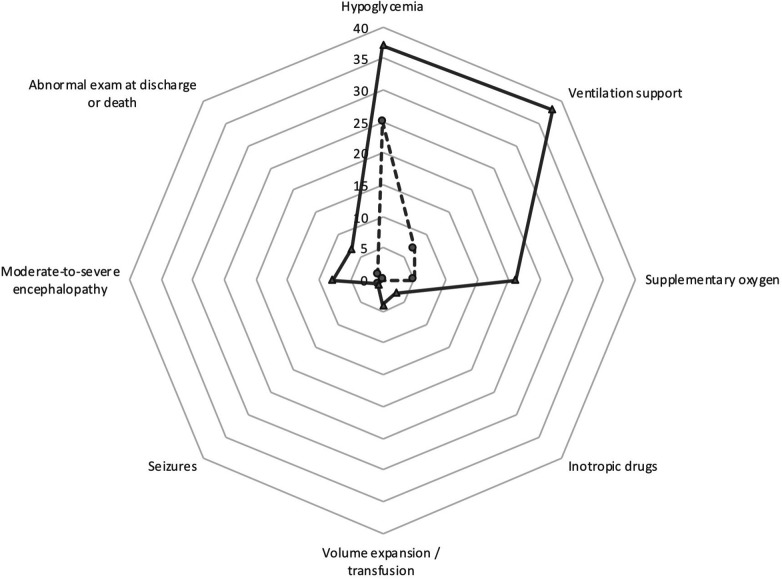
Main comorbidities of the cohort study. The data for the pH ≤7.00 cohort are shown in solid line and the data for the pH 7.01–7.10 cohort are shown in dashed line.

Regarding comorbidities within 6 h of life (Table 2, Figure 2), infants with ApH ≤7.00 needed respiratory support more often than did infants with ApH 7.01–7.10. This was either mechanical ventilation (13% vs. 1%), non-invasive ventilation (26% vs. 7%), or supplementary oxygen needs (21% vs. 5%) (*p* < 0.001). Ventilation support was 5.1 times (95%CI 2.87, 9.03; *p* < 0.001) more likely to be needed by those with cord ApH ≤7.00 compared to those with ApH 7.01–7.10.

**Table 2:** Neonatal morbidity within 6 h of postnatal life.

Variables	Total (*N* = 293)	Cord pH 7.01–7.10 (*N* = 176)	Cord pH ≤7.00 (*N* = 117)	*P*-value	OR (95% CI)
Any neurological or systemic involvement^a^	88/293 (30)	12/176 (28)	76/117 (65)	0.000	9.53 (5.43, 16.71)
Severe neurological or systemic involvement^b^	16/293 (6)	2/176 (1)	24/117 (13)	0.000	18.05 (4.35, 74.94)
Moderate or severe HIE	9/293 (3)	0	9/117 (8)	0.000	–
No HIE	264/293 (90)	165/176 (94)	99/117 (85)	0.001	
Mild HIE	20/293 (7)	11/176 (6)	9/117 (8)		1.33 (0.57, 3.11)
Moderate HIE	6/293 (2)	0	6/117 (5)		–
Severe HIE	3/293 (1)	0	3/117 (3)		–
Seizures	2/293 (1)	1/176 (1)	1/117 (1)	1.000	1.50 (0.10, 23.81)
Passive cooling	92/286 (32)	5/176 (3)	87/110 (79)	0.000	27.84 (11.67, 66.41)
Any systemic involvement^c^	130/293 (44)	53/176 (30)	77/117 (66)	0.000	9.53 (5.43, 16.71)
Severe systemic involvement^d^	18/293 (6)	2/176 (1)	16/117 (14)	0.000	12.03 (2.82, 51.37)
Supplementary oxygen	33/293 (11)	8/176 (5)	25/117 (21)	0.000	4.70 (2.20, 10.06)
Non-invasive or mechanical ventilation	57/293 (19)	13/176 (7)	44/117 (38)	0.000	5.09 (2.87, 9.03)
Non-invasive ventilation	42/293 (14)	12/176 (7)	30/117 (26)	0.000	3.76 (2.01, 7.04)
Mechanical ventilation	17/293 (6)	2/176 (1)	15/117 (13)	0.000	11.28 (2.63, 48.42)
Need of inotropic drugs	4/293 (1)	0/176	4/117 (3)	0.025	–
Volume expansion/transfusion	5/293 (2)	0/176	5/117 (4)	0.010	–
Hypoglycemia (<46 mg/dl)	85/283 (30)	42/168 (25)	43/115 (37)	0.026	1.50 (1.05, 2.13)
Hours of life to hypoglycemia, median (IQR)	1 (1, 3)	1 (1, 1)	1 (1, 3)	0.013	–
Glucose intake (oral or IV) previous to diagnosis of hypoglycemia	47/85 (55)	32/42 (76)	15/43 (35)	0.000	
IV glucose shortly after admission after birth	62/293 (21)	10/176 (6)	52/117 (44)	0.000	7.82 (4.14, 14.76)
IV glucose administration within 6 h of life	85/293 (29)	18/176 (10)	67/117 (57)	0.000	5.60 (3.52, 8.91)
Abnormal exam at discharge or death	9/293 (3)	1/176 (1)	8/117 (7)	0.003	12.03 (1.53, 94.96)
Death	2/193 (1)	0/176	2/117 (2)	0.159	
Abnormal clinical exam (at discharge) in surviving infants	7/291 (3)	1/176 (1)	6/115 (5)	0.009	9.18 (1.12, 75.28)

Categorical variables are expressed in *n*/*N* (%). HIE, hypoxic-ischemic encephalopathy; IQR, interquartile range; RR, relative risk; IV, intravenous.

^a^ Any neurological or systemic involvement denotes the presence of at least one of the following: (a) seizures, (b) any degree of NE, (c) any systemic involvement (see below).

^b^ Severe neurological or systemic involvement denotes the presence of seizures, (b) moderate or severe encephalopathy, (c) severe systemic involvement (see below).

^c^ Any systemic involvement includes the presence of at least one of the following: (a) any respiratory support (supplementary oxygen, non-invasive, and/or mechanical ventilation), (b) need for inotropic drugs, (c) need for volume expansion or transfusion, (d) hypoglycemia <46 mg/dl.

^d^ Severe systemic involvement includes the presence of at least one of the following: (a) mechanical ventilation, (b) need of inotropic drugs, (c) volume expansion or transfusion.

A few infants with ApH ≤7.00 needed hemodynamic support with inotropes (4 infants) and/or volume expansion/transfusions (5 infants), whereas none of the infants with ApH 7.01–7.10 did (*p* < 0.05).

Hypoglycemia (< 46 mg/dl) occurred in both cohorts, though it was more frequent in infants with ApH ≤7.00 compared to those with ApH 7.01–7.10 (37% vs. 25%; *p* = 0.026), and it was at a later age (*p* = 0.013): it happened at 1 and 3 h of life in 39% and 81% infants with ApH ≤7.00, respectively, compared to 61% and 19% in the 7.01–7.10 ApH cohort. Intravenous glucose was started shortly after admission in almost half of the infants with ApH ≤7.00. In 55% of the infants in the entire study cohort had hypoglycemia despite oral (breastfeeding) or intravenous glucose administration was initiated (Table 2).

Regression analysis showed that none of the perinatal variables that were found to be statistically different in the univariate analysis were associated independently with the categorization of ApH in ≤7.00 and 7.01–7.10. However, same analyses including the variable moderate-to-severe HIE showed an OR of 41.83 (95% CI 2.94, 596.08; *p* = 0.006) for chorioamnionitis, and 20.92 (95% CI 1.77, 247.12; *p* = 0.016) for general anesthesia.

## 4 Discussion

The results of this study support the hypothesis that the incidence of HIE is more prevalent in newborns with pH ApH ≤7.00 than in those who had ApH 7.01–7.10. All infants with moderate or severe NE were in the ApH group ≤7.00, which supports the premise that surveillance programs for detecting TH candidates should be aimed at infants with cord pH ≤7.00.

Comorbidity in patients with perinatal asphyxia is relatively well known, but few studies have focused on what happens in the first 6 h of life. This study reflects the non-negligible comorbidity in this population, and warns of the incidence of hypoglycemia, not only in the ApH ≤7.00 cohort, but also in the 7.01–7.10 cohort.

The development of programs in neonatal units to establish which newborns with perinatal asphyxia should be monitored most closely during the first hours of life is not trivial. Identifying the newborn with HIE and establishing the severity of this is crucial to controlling comorbid factors during the first hours of life, in order to delineate management strategies, including TH, but also to plan the services involved in long-term follow-up. In fact, early identification of neonates with moderate or severe HIE is one of the strategies to optimize early initiation of HT and maximize its neuroprotective effect, improving outcomes (2, 5, 6, 38, 39). There are many studies that have analyzed the relationship between perinatal acidosis and neonatal encephalopathy, as well as other clinical morbidities and long-term adverse outcomes. However, inclusion criteria based on the cord pH threshold and rate of infants in whom cord gas is performed, as well as the definition and age of encephalopathy ascertainment, are heterogeneous among the studies (15, 40).

A relevant question is what pH threshold might be advisable to be set in a perinatal surveillance program to detect NE, especially moderate cases in which the benefits of TH are greatest (41). The first clinical trials established the cord pH cut-off point at 7.00 (42, 43), although there are infants with moderate-to-severe HIE above this value in all TH series (1, 18, 22, 25, 36, 44, 45). These series, however, analyze cohorts of HIE patients, but they do not include all consecutively born infants with perinatal acidemia.

Studies in the past warned that as the pH at birth decreases, the risk for neonatal encephalopathy and neurological comorbidity increases (15, 40, 46). One meta-analysis showed that low cord ApH was significantly associated with neonatal encephalopathy, up to an OR of 13.8 (CI 6.6 to 28.9, *I*^2 ^= 0%) (15). However, though the threshold pH 7.00 was at the highest risk for neonatal encephalopathy, association was also present above that level (15, 40).

In fact, metabolic acidosis in umbilical arterial blood is the most objective assessment of the presence of intrapartum hypoxia-ischemia and it is quite universally agreed upon that cord pH threshold of 7.00 is a good cut-off point to define intrapartum hypoxia-ischemia. However, there is no single umbilical ApH value that clearly separates those infants who will or will not have HIE (40).

One of the most recent large studies, though with a sampling rate of half of the births, showed that moderate or severe HIE occurred in 8.5% infants with ApH <7.00 but also in 1.2% of those with ApH 7.00–7.10 (47). However, another study, with also half cord sampling, reported 1.2% and 0.2% of encephalopathy with seizures within 24 h of life among infants with ApH ≤7.00 and 7.01–7.10, respectively. Nevertheless, the definition of HIE, its severity, and its age of ascertainment in the two studies were at odds (15, 16, 47, 48).

On the other hand, despite severe acidemia, most infants will still recover fully without notable illness, so the question arises as to whether they should be admitted to NICU and therefore benefit from closer surveillance, or whether it is preferable to leave them with their parents to promote skin-to-skin care and parent-child emotional bonding. While 6.8% and 1.2% of infants in our study had pH ≤7.10 and pH ≤7.00, respectively, and while these percentages may not seem relevant, they do represent a high number of infants to be monitored in units with a high number of births, leading to a debate as to whether admission is a cost-effective and beneficial measure in a surveillance program.

Since the introduction of TH as the standard of treatment for moderate or severe HIE has narrowed the time frame for establishing the diagnosis, these golden hours are crucial to initiate the hypothermia code that includes stabilization and close monitoring that includes gas analysis, surveillance of comorbid factors such as temperature, glucose, cardiovascular and respiratory function, and obviously determination of the presence and severity of HIE in order to start TH (33, 39, 49, 50). Due to the need to diagnose and establish the severity of HIE and monitor the associated multiorgan dysfunction, we agree with other authors that recommend close monitoring of infants with severe acidemia who appear clinically well immediately after delivery for up to 24 h, ideally with concomitant aEEG (51).

Our study notes that all patients had moderate or severe HIE in the ApH ≤7.00 cohort. This would allow a cut-off point of 7.00 to be established as a safe value to delimit which children should be monitored most closely. Furthermore, given that there is a non-negligible number of children with pH ≤7.00 who develop respiratory distress requiring respiratory support, with some patients requiring cardiovascular support, this reinforces the idea that this group of children could be eligible for admission to the NICU during those first 6 h after the hypoxic-ischemic event.

However, a not insignificant number of infants with pH 7.01–7.10 showed non-negligible comorbidities during the first 6 h, including those with hypoglycemia and those that required respiratory support. In addition, 6% of these infants were diagnosed of mild HIE. An important fact to highlight from the study is that two thirds of the patients showed hypoglycemia values in spite of being either on oral supplementation (mostly infants with pH 7.01–7.10 who were left with their breastfeeding mother) or intravenous supplementation in the form of glucose solution (mostly infants with pH ≤7.00). Following a hypoxic-ischemic insult, the brain is particularly vulnerable to early comorbid factors with the potential to damage the central nervous system, shorten the therapeutic window, and increase the severity of the injury. Plasma glucose level shows a negative correlation with severity of HIE (28, 29). More importantly, hypoglycemia in the first 6 h is consistently associated with death or disability at 18–24 months (29–31). This association is independent of Apgar, severity of HIE, and TH (30, 31). An unexpected finding was that in our cohort, a quarter of the infants with ApH ≤7.10 had hypoglycemia within 6 h of life, and although this happened more often in those with ApH ≤7.00, our results suggest that both groups of infants require close and rigorous blood glucose monitoring during the first 6 h. Since infants in the ApH 7.01–7.10 group are not admitted to the NICU, nor are they started on intravenous glucose solution, we do not know if a pattern similar to that for infants with ApH ≤7.00 would have prevented episodes of hypoglycemia.

It is important to note that the findings of this study are aimed at programs with close monitoring during the first 6 h of life since both cohorts were clinically monitored serially with various exams and with glycemia within the first 6 h of life. We cannot rule out the possibility that if this monitoring had not been carried out, the altered outcome rates may have been different, with greater comorbidity or even aggravation of the HIE, not only in the ApH ≤7.00 group but also in the cohort with ApH 7.01–7.10. In addition, the performance of various exams in the first 6 h may have helped to identify cases with moderate HIE in whom altered alertness may be less easily recognized than in those with severe HIE (52).

It has been well documented that the risk of neonatal mortality increases as the pH at birth decreases (15, 40). Though our morbidity percentages were lower than in other studies, possibly due to a high level of umbilical cord gas data, it is notable that developing moderate-to-severe HIE in our cohort was a risk factor of greater impact for mortality and adverse neurological outcome at discharge than was the ApH value itself.

Though infants in our study were not included on the basis of their base deficit values, our cohort showed a strong correlation between base deficit and ApH values, and abnormal base deficit did not predict the presence of HIE or other clinical abnormalities better than did pH values. It seems that pH cord values, and not base deficit levels, are the parameter most closely associated with outcome, and base deficit adds no further predictive value above ApH alone (16, 53).

Several limitations of the present study should be acknowledged. First it is from one center. Second, other inclusion criteria at birth, including abnormal base deficit and lactate, might have added more predictive value than pH alone in detecting those infants who would present HIE. However, after reviewing all infants diagnosed with moderate or severe HIE during the study period, there was only one infant out of this cohort who had an ApH of 7.11 and base deficit of 6.9 mmol/L.

Third, our study did not establish a strict criterion under which hypoglycemia should initially be corrected either with oral inputs or intravenous therapy. Thus, we cannot identify which approach would be the safest and most efficient to protect infants with perinatal acidemia from hypoglycemia, or whether the approach would differ depending on whether the ApH was less than 7.00 or higher.

Fourth, it is known that there are other factors that may shorten the therapeutic window and increase the severity of the damage after a hypoxic-ischemic event, such as hyperthermia and hypotension. Children at pH 7.01–7.10 were not separated from their parents, so close monitoring to measure these variables as they were applied to newborns with pH ≤7.00 was not possible. In fact, most infants with cord pH ≤7.00 (79% in our series) underwent passive cooling measures to prevent hyperthermia or to set mild hypothermia until the decision on the need for TH was made. We cannot rule out an interaction effect of this approach on the results of the glycemia tests and the other clinical outcomes.

Fifth, due to the low incidence of moderate and severe HIE, our study does not have enough power to determine whether other factors added to the cord pH value might have predictive ability for HIE development in the first 6 h of life.

Strengths of the present study included the large size of the cohort with complete umbilical cord gas collection data, and the systematic grading of HIE throughout several exams within the first six hours, supported by a previous educational program.

In conclusion, the present study findings indicate that cord pH 7.00 might be a safe pH cut-off point when developing protocols to monitor infants born with acidemia in order to identify infants with moderate or severe HIE early on, and they argue for surveillance of comorbid factors, especially hypoglycemia, which can occur in the first 6 h of life mostly in infants with cord pH ≤7.00, but also in those with pH 7.01–7.10. These results have implications in the management of local and regional programs for the surveillance of infants with perinatal acidemia who are candidates for neuroprotection treatments, and they may lead to early identification of HIE as well as to more efficient implementation of surveillance of early comorbid factors and the establishment of preventive measures.

## Data Availability

The raw data supporting the conclusions of this article will be made available by the authors, without undue reservation.
